# Comparative Prognostic Roles of β-Catenin Expression and Tumor–Stroma Ratio in Pancreatic Cancer: Neoadjuvant Chemotherapy vs. Upfront Surgery

**DOI:** 10.3390/curroncol32100578

**Published:** 2025-10-17

**Authors:** Shu Oikawa, Hiroyuki Mitomi, So Murai, Akihiro Nakayama, Seiya Chiba, Shigetoshi Nishihara, Yu Ishii, Toshiko Yamochi, Hitoshi Yoshida

**Affiliations:** 1 Department of Medicine, Division of Gastroenterology, Showa Medical University School of Medicine, Tokyo 142-8555, Japan; a.nakayama@med.showa-u.ac.jp (A.N.); s.nishi@med.showa-u.ac.jp (S.N.); yu580823@med.showa-u.ac.jp (Y.I.); hyoshida@med.showa-u.ac.jp (H.Y.); 2 Department of Diagnostic Pathology, Showa Medical University School of Medicine, Tokyo 142-8555, Japan; hmitomi@med.showa-u.ac.jp (H.M.); smurai1015@med.showa-u.ac.jp (S.M.); seiyachiba@med.showa-u.ac.jp (S.C.); onizuka@med.showa-u.ac.jp (T.Y.); 3 Department of Gastroenterological Surgery, Showa Medical University School of Medicine, Koto Toyosu Hospital, Tokyo 135-8577, Japan

**Keywords:** β-catenin, pancreatic ductal adenocarcinoma, digital image analysis, immunohistochemistry, neoadjuvant chemotherapy, tumor budding, tumor–stroma ratio, upfront surgery, whole-slide imaging

## Abstract

The benefit of neoadjuvant chemotherapy (NAC) over upfront surgery (UFS) for resectable pancreatic ductal adenocarcinoma (PDAC) is increasingly recognized, yet prognostic biomarkers remain undefined. We evaluated tumor–stroma ratio (TSR), β-catenin (β-CTN) expression, and tumor budding (TB) in 84 resected PDACs (35 NAC, 49 UFS) using digital image analysis of multi-cytokeratin (m-CK) and β-CTN immunohistochemistry. TSR was defined as the proportion of malignant epithelial area within the tumor, and the β-CTN/m-CK index as the ratio of β-CTN to m-CK immunoreactivity in tumor tissue relative to intralobular ducts. TB was significantly less frequent in NAC than UFS (*p* = 0.003), suggesting that NAC may indirectly modulate epithelial–mesenchymal transition, with TB regarded as its morphological correlate. In the NAC cohort, low TSR was associated with more favorable histological response (Evans IIa/IIb, median 7%; Evans I, 16%; *p* = 0.009), likely reflecting NAC-induced tumor shrinkage with relative stromal predominance. In multivariable analysis, low β-CTN/m-CK index (<0.5) predicted shorter relapse-free survival in both NAC (HR = 2.516, *p* = 0.043) and UFS (HR = 2.230, *p* = 0.025) subgroups. High TSR (≥13%) was associated with shorter cancer-specific survival (HR = 2.414, *p* = 0.034) in the overall cohort, indicating prognostic value complementing its association with NAC response. These results identify the β-CTN/m-CK index and TSR as prognostic biomarkers in PDAC.

## Simple Summary

Pancreatic cancer is one of the most aggressive cancers, and doctors need better ways to estimate which patients may relapse after surgery. In this study, we analyzed tissue samples from 84 patients, some who received chemotherapy before surgery and some who went straight to surgery. Using computer-based imaging, we looked at several microscopic features. First, we measured β-catenin, a protein that helps cells stick together, and found that patients with low levels were more likely to experience cancer recurrence. We also examined the tumor–stroma ratio (TSR), the balance between cancer cells and surrounding supportive tissue, which showed important links with treatment response and survival. Finally, we evaluated tumor budding, small groups of cells that indicate invasive growth, and found that chemotherapy before surgery reduced this feature. These results suggest that simple microscopic tests can help predict outcomes and guide more personalized treatment for people with pancreatic cancer.

## 1. Introduction

The incidence of pancreatic cancer (PC) has increased over the past few decades, accounting for 2–3% of all cancers [1,2], and is highest in North America and Europe, followed by East Asia [3]. PC causes about 5% of cancer deaths worldwide [2,3], with an extremely poor 5-year survival rate of 2–13% [1,2]. For localized disease, surgical resection remains the main curative option [1,2,3]. The 5-year survival after surgery has improved to ~15–25% [3], up from 1.5% to 17.4% between 1975 and 2011 [2].

Neoadjuvant chemotherapy (NAC) may enhance outcomes by eradicating occult metastases and increasing resection rates [1,2], though its survival benefit remains debated [4]. A meta-analysis of 11 studies reported similar survival between resectable PC with or without NAC [5], while a retrospective study of 1175 NAC and 4041 upfront surgery (UFS) cases showed improved survival with multiagent NAC [6]. Phase III trials in Japan established S-1, an oral prodrug of 5-fluorouracil, as an effective and convenient alternative to infusion therapy [7]. In PC, S-1 demonstrated comparable or superior efficacy to gemcitabine (GEM) in advanced and adjuvant settings [4]. The Japanese multicenter PREP-02/JSAP-05 trial showed that NAC with S-1 plus GEM significantly improved survival in resectable PC [8], and this regimen is now recommended by the Japan Pancreas Society [9]. Although S-1 has shown promising efficacy in Caucasian populations [10], its use in Western countries is limited by lower tolerated doses due to gastrointestinal toxicity [4,7]. While NAC is integral to domestic treatment, interest is increasing in histopathological assessment of residual tumor aggressiveness as prognostic markers.

Pancreatic ductal adenocarcinoma (PDAC) is the predominant subtype [1,3], characterized by marked heterogeneity and a dense stroma comprising up to ~80–90% of tumor volume, which influences tumor progression and drug delivery [11,12]. PDAC generally exhibits a lower tumor–stroma ratio (TSR) than other cancers [13]. The prognostic value of TSR has been demonstrated across malignancies [13] and validated in PDAC [14,15,16,17,18,19,20,21,22]. Computer-aided image analysis has enabled more objective TSR quantification [15,17,18,19,20,21,22,23,24,25]. Evidence suggests that the stromal compartment mediates chemoresistance in PC [26,27,28,29]. Experimental models show that stromal depletion enhances drug delivery [28,29], and S-1–based regimens reduce stroma and induce apoptosis [29,30]. However, the prognostic relevance of TSR in PDAC treated with NAC remains poorly defined [18,20].

β-catenin (β-CTN) anchors cadherin complexes and mediates Wnt signaling [31]. Loss of β-CTN disrupts adhesion and promotes invasion, with lower expression in poorly differentiated tumors [32]. Cytoplasmic localization of β-CTN correlates with advanced disease and poor prognosis [33,34,35,36], while overexpression may promote chemoresistance [37]. Despite its importance, only limited histological parameters, such as encapsulating fibrosis [20], β-CTN/PROX1 co-expression [34], podocalyxin [38], and GATA6 [39], have been systematically investigated.

Tumor budding (TB), defined as single cells or small clusters of ≤4 cells at the invasive front, reflects epithelial–mesenchymal transition [40,41,42] and is associated with stromal and immune components [43]. Although TB is an independent prognostic factor in PDAC [42,44,45,46,47,48], its significance after NAC remains underexplored [44,45].

Against this background, we investigated TSR, β-CTN expression, and TB in resectable PDAC, analyzed their clinicopathological associations, and evaluated their prognostic relevance, particularly in NAC versus UFS cohorts.

## 2. Materials and Methods

### 2.1. Patients and Materials

This retrospective study included 84 consecutive Japanese patients with PDAC who underwent curative-intent resection at Showa Medical University Hospital between January 2018 and December 2023. Patients with other histologies (e.g., acinar cell carcinoma, neuroendocrine neoplasms) were excluded. Patients with borderline-resectable PDAC and those undergoing conversion surgery, as previously defined [49,50], were also excluded. Of the 84 resectable cases, 35 received NAC consisting of two courses of S-1 (body-surface area < 1.25 m^2^, 80 mg/day; 1.25–1.4 m^2^, 100 mg/day; ≥1.5 m^2^, 120 mg/day) for 14 consecutive days followed by a 1-week rest, plus GEM (1000 mg/m^2^ on days 1 and 8 followed by a 2-week rest), and then underwent pancreatectomy (NAC group). The remaining 49 underwent UFS with no preoperative therapy (UFS group). A flow diagram illustrating patient selection is provided in Supplementary Figure S1. Adjuvant S-1 (four 28-day courses with a 2-week rest) was administered to 48/84 patients. The mean follow-up among survivors at last visit (*n* = 48) was 732 days (range, 59–1899). Clinicopathological data are summarized in Supplementary Table S1. Written informed consent was obtained from all patients. The study was approved by the Showa Medical University Ethics Committee (No. 2024-233-B) and conducted in accordance with the Declaration of Helsinki.

### 2.2. Histological Analysis

Resected specimens were fixed in 10% neutral-buffered formalin, sliced at 5 mm intervals, and embedded in paraffin. Tissue sections, 4 µm in thickness, were stained with hematoxylin and eosin (H&E). Two pathologists (H.M., S.M.), blinded to clinical data, reviewed all H&E slides. Pathological T and N categories and overall stage were assigned according to the UICC TNM classification (9th edition). The histological effect of NAC was graded by the Evans system [51], as follows: Grade I, little or no tumor cell destruction (<10%); Grade IIa, 10–50%; Grade IIb, 51–90%; Grade III, >90% with only single cells or small clusters remaining; Grade IV, no viable tumor cells. For IHC, one representative block from the largest tumor slice including adjacent pancreatic tissue was selected.

### 2.3. Immunohistochemstry (IHC)

From each representative block, serial sections (4 μm) were cut and placed on three slides; two for IHC and one for H&E. After heat-induced epitope retrieval (BOND Epitope Retrieval Solution 1, Leica Biosystems), slides were immunostained on an automated stainer (BOND-III, Leica Biosystems, Newcastle upon Tyne, UK) with mouse monoclonal multi-cytokeratin (m-CK) (clone AE1/AE3, 1:200; Leica Biosystems, Newcastle upon Tyne, UK) and monoclonal β-CTN (clone 17C2, prediluted; Leica). Detection used a horseradish peroxidase-labeled polymer (BOND compact polymer system). Immunoreactivity was visualized with 3, 3′-diaminobenzidine and counterstained with hematoxylin.

### 2.4. TSR

Whole slide images (WSIs) of H&E and m-CK IHC were acquired at ×40 using the NanoZoomer S60 (Hamamatsu Photonics, Hamamatsu, Japan) and saved as non-layered JPEGs. Using ImageJ 1.54g (National Institutes of Health, Bethesda, MD, USA), tumor areas were annotated on m-CK WSIs with reference to serial H&E WSIs and reviewed in NDP.view2 (v2.9.29). Briefly, the tumor area on the m-CK WSI (corresponding to H&E) was outlined; non-tumorous surrounding tissue and intra-tumoral non-neoplastic parenchyma were removed; images were converted to 8-bit grayscale and then to binary. Annotation was performed by one observer (S.O.) and reviewed by a pathologist (H.M.). The lower threshold was set to zero and the upper threshold fixed at 140 across cases to suppress background while preserving true immunoreactivity. In the final binary image, m-CK-positive tumor area (white) and m-CK-negative stromal area (black) were quantified, and TSR (%) was calculated as:

$$TSR(\%)=\frac{m\text{-}\mathrm{CK}\text{-}\mathrm{positive}\;\mathrm{tumor}\;\mathrm{area}\;[{\mathrm{mm}}^{2}]}{m\text{-}\mathrm{CK}\text{-}\mathrm{positive}\;\mathrm{tumor}\;\mathrm{area}\;[{\mathrm{mm}}^{2}]+m\text{-}\mathrm{CK}\text{-}\mathrm{negative}\;\mathrm{stromal}\;\mathrm{area}\;[{\mathrm{mm}}^{2}]}\times 100$$

**Figure 1:**
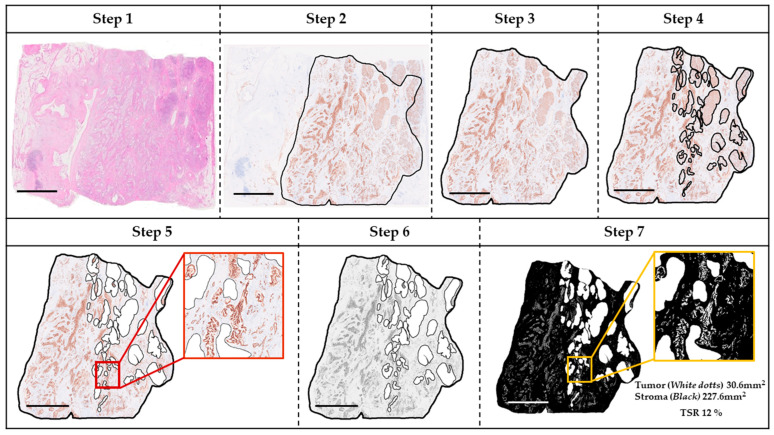
Representative example of TSR analysis. Step 1: Tumor area scanned in hematoxylin and eosin staining. Step 2: Tumor area outlined by m-CK immunohistochemistry. Step 3: Surrounding non-tumorous areas eliminated. Step 4: Non-tumorous regions within the tumor mass delineated. Step 5: Zoomed view showing eliminated non-tumorous areas and m-CK–stained tumor regions. Step 6: Construction of gray-scale image. Step 7: Construction of binary image with a zoomed view of the boxed region. Black and white areas correspond to stroma (non-tumorous parenchyma) and tumor, respectively. Tumor and stroma areas were 30.6 mm^2^ and 227.6 mm^2^, respectively, yielding a TSR (= tumor/[tumor + stroma] × 100) of 12%. Scale bar = 5 mm. Abbreviations: TSR, tumor–stroma ratio; m-CK, multi-cytokeratin.

The workflow is illustrated in Figure 1; ImageJ settings are detailed in the Supplementary Methods.

### 2.5. Assessment of β-CTN IHC

Nuclear/cytoplasmic and membranous β-CTN stainings were assessed separately. Distinct nuclear and/or cytoplasmic staining was considered positive regardless of intensity and dichotomized as negative (<1% positive cells) or positive (≥1%). Slides were assessed concurrently by two authors (S.O., H.M.) using a multi-head microscope, blinded to outcomes; disagreements (<10% of cases) were resolved by joint review.

### 2.6. β-CTN/m-CK Index

WSIs of serial H&E, β-CTN, and m-CK stained slides were scanned, and five representative images of tumor and adjacent intralobular ducts were captured per case at ×5–10 magnification and saved as non-layered JPEGs. Using ImageJ, β-CTN and m-CK images were converted to 8-bit grayscale, and thresholds for binary conversion (black and white) were determined by two observers, including a pathologist (S.O. and H.M.). The maximum threshold was fixed at 140 across all analyses. In the resulting binary images, white areas corresponded to β-CTN-positive or m-CK-positive regions. The β-CTN/m-CK index was calculated as the ratio of β-CTN/m-CK in the tumor normalized to the corresponding ratio in intralobular ducts:

$$\beta \text{-}\mathrm{CTN}/m\text{-}\mathrm{CK}\;\mathrm{index}=\frac{\mathrm{Ratio}\;\mathrm{of}\;\beta \text{-}\mathrm{CTN}\;\mathrm{stained}\;\mathrm{area}\;\mathrm{to}\;m\text{-}\mathrm{CK}\;\mathrm{stained}\;\mathrm{area}\;\left[{\mathrm{mm}}^{2}\right]}{\mathrm{Ratio}\;\mathrm{of}\;\beta \text{-}\mathrm{CTN}\;\mathrm{stained}\;\mathrm{intralobular}\;\mathrm{area}\;\mathrm{to}\;m\text{-}\mathrm{CK}\;\mathrm{stained}\;\mathrm{intralobular}\;\mathrm{area}\;\left[{\mathrm{mm}}^{2}\right]}$$

**Figure 2:**
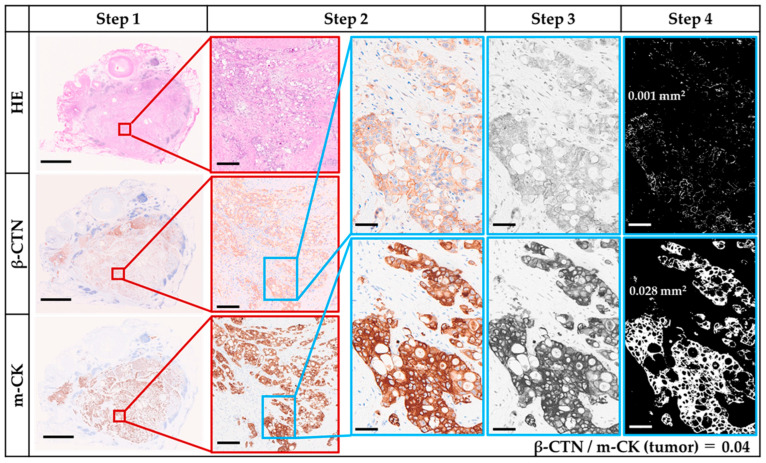
Illustration of β-CTN/m-CK in a low-index (<0.5) case. Step 1: Scanning of the tumor (scale bar = 5 mm). Step 2: Capture of representative image (scale bar = 200 µm) with higher magnification of the boxed region (scale bar = 50 µm). Step 3: Construction of gray-scale image (scale bar = 50 µm). Step 4: Construction of binary image (scale bar = 50 µm). White areas corresponding to tumor were measured as 0.001 mm^2^ in β-CTN and 0.028 mm^2^ in m-CK images, yielding a β-CTN/m-CK ratio of 0.04 for the tumor. The same stepwise analyses were applied to the non-neoplastic intralobular duct (shown in supplementary Figure S2). The final β-CTN/m-CK index was 0.4. Abbreviations: β-CTN, β-catenin; m-CK, multi-cytokeratin.

Stepwise analysis for a representative case with β-CTN/m-CK index < 0.5 is illustrated in Figure 2. Step 1: Tumor regions were scanned on β-CTN and m-CK WSIs corresponding to serial H&E sections. Step 2: Five representative tumor images were captured at ×10 magnification. Step 3: Grayscale (8-bit) images were generated. Step 4: Binary images were created, in which white areas represent β-CTN- or m-CK-positive tumor regions. Step 5: The β-CTN/m-CK ratio was calculated for each tumor image. The same procedure was applied to non-neoplastic intralobular ducts (Supplementary Figure S2). Representative analysis of a case with β-CTN/m-CK index ≥ 0.5 is shown in Supplementary Figure S3.

### 2.7. TB

TB was assessed on m-CK IHC slides to improve detectability. Following the consensus definition [41], TB was defined as single tumor cells or clusters of up to four cells without connection to the main tumor mass. Two authors (S.O., H.M.) screened the entire slide at low magnification to identify the hot spot (densest TB area), irrespective of location. TBs within the hot spot were counted at ×20 objective (field area 0.785 mm^2^). TB was dichotomized as low (<10 buds) or high (≥10 buds). TB was dichotomized as low (<10 buds) or high (≥10 buds), with the cutoff determined using the Contal–O’Quigley/maxstat method described below. Representative examples are provided in Supplementary Figure S4.

### 2.8. Statistical Analysis

Categorical variables were compared with the χ^2^ test or Fisher’s exact test; continuous variables with the Mann–Whitney U test. Spearman’s rho was used to assess non-parametric correlations. Cancer-specific survival (CSS) was measured from surgery to death from PDAC or last follow-up; deaths from other causes were censored. Relapse-free survival (RFS) was measured from surgery to the first event of locoregional recurrence, distant metastasis, or death from any cause. Kaplan–Meier curves were generated and compared using the log-rank test (and, where indicated, Wilcoxon). Multivariate analyses used Cox proportional hazards models with stepwise selection; candidate variables entered if univariate *p* < 0.10. For threshold determination, the maximally selected log-rank method (Contal–O’Quigley/maxstat) was applied to identify the cutoff yielding the smallest log-rank *p*-value around the median while maintaining balanced group sizes. Hazard ratios (HRs) with 95% confidence intervals (CIs) were reported. Two-sided *p* < 0.05 was considered statistically significant. Analyses were performed using JMP pro version 16 (SAS Institute, Cary, NC, USA).

## 3. Results

### 3.1. Clinicopathological Differences Between NAC and UFS

Patients in the UFS group were significantly older than those in the NAC group (*p* = 0.040). The distribution of pT category approached significance (*p* = 0.060); the NAC cohort included more pT1 and fewer pT2, with a modest excess of pT3 (NAC: pT1 13/35, 37.1%; pT2 13/35, 37.1%; pT3 9/35, 25.7% vs. UFS: pT1 10/49, 20.4%; pT2 31/49, 63.3%; pT3 8/49, 16.3%). Margin status likewise showed a borderline difference (*p* = 0.067), with margin-negative resections being more frequent after NAC (33/35, 94.3%) than UFS (39/49, 79.6%) and margin-positive resections correspondingly less frequent (NAC 2/35, 5.7% vs. UFS 10/49, 20.4%). In contrast, TSR and the β-CTN/m-CK index did not differ between groups (Table 1). Kaplan–Meier analysis revealed no significant difference in CSS or RFS between NAC and UFS.

### 3.2. β-CTN and m-CK IHC

In non-neoplastic pancreas, β-CTN was distinctly membranous in intralobular ducts but weak in acinar cells. In PDAC, tumors largely retained membranous expression, yet loss of membranous staining became evident with poor differentiation. Nuclear and cytoplasmic staining was uniformly absent (<1% of tumor cells). In contrast, m-CK showed uniformly strong staining across tumor and non-neoplastic components, largely preserved irrespective of differentiation.

### 3.3. Baseline Values of TSR, β-CTN/m-CK Index, and TB

The median TSR was 14% (range, 2–53) in the entire cohort, with similar values in the NAC (12%, range 2–51) and UFS groups (14%, range 2–53). The β-CTN/m-CK index showed a median of 0.5 (range, 0–12.3) overall, tending to be higher in the NAC group (0.8, range 0–12.3) compared with the UFS group (0.5, range 0–5.2). The median TB count per hotspot (×20, 0.785 mm^2^) was 15.5 (range, 1–250) overall, 10 (range, 1–250) in the NAC group, and 17 (range, 4–90) in the UFS group. Low TB was observed in 27% of cases (46% in NAC vs. 14% in UFS), while high TB (73% of cases) predominated (54% in NAC vs. 86% in UFS).

### 3.4. Association of TSR with Clinicopathological Features

Patients were dichotomized by TSR at 13% (pre-specified and approximating the cohort median) into <13% and ≥13% groups. The cutoff was determined using the Contal–O’Quigley/maxstat method. Resection margin status showed a marginal trend with TSR in the overall cohort (*p* = 0.058): margin-negative resections were 35/37 (94.6%) in the TSR < 13% group versus 37/47 (78.7%) in the TSR ≥13% group. A similar pattern was observed in the UFS subgroup (*p* = 0.070), with margin-negative resections 17/18 (94.4%) for TSR < 13% compared with 22/31 (71.0%) for TSR ≥13% (Supplementary Table S2).

### 3.5. Association of β-CTN/m-CK Index with Clinicopathological Features

Patients were categorized into two groups according to the β-CTN/m-CK index (<0.5 vs. ≥0.5). This cutoff was likewise defined using the Contal–O’Quigley/maxstat method. Overall, the index showed no significant associations with clinicopathological variables. Within the NAC subgroup, however, high index (≥0.5) was more frequent in younger (<75 years) than in older patients (younger 17/22, 77.3% vs. older 6/13, 46.2%; *p* = 0.079) (Supplementary Table S3). Although this dichotomization was not associated with tumor differentiation, stratified analysis showed significantly lower index values in poorly compared with well-differentiated tumors (median 0.3 vs. 0.9; *p* = 0.046).

### 3.6. Association of TB with Clinicopathological Features

In the overall cohort, peripancreatic fat invasion was more frequent with high vs. low TB (53/61, 86.9% vs. 14/23, 60.9%; *p* = 0.014); perineural invasion and moderate/poor differentiation showed similar trends (50/61, 82.0% vs. 14/23, 60.9%; *p* = 0.082; 38/61, 62.3% vs. 9/23, 39.1%; *p* = 0.084). In the NAC subgroup, no association reached significance, though moderately/poorly differentiated tumors tended to show higher TB (14/19, 73.7% vs. 7/16, 43.8%; *p* = 0.094). In the UFS subgroup, age ≥ 75 years and peripancreatic fat invasion were enriched in the high-TB group (25/42, 59.5% vs. 0/7, 0.0%; *p* = 0.004; 38/42, 90.5% vs. 3/7, 42.9%; *p* = 0.009). No other variables showed significant or marginal associations (Supplementary Table S4).

### 3.7. Interrelationships Among β-CTN/m-CK Index, TSR, and TB in PDAC

By Spearman’s rank correlation, the β-CTN/m-CK index showed a weak inverse association with TB in the overall cohort (*rho* = –0.320, *p* = 0.003), which was consistently recapitulated in the UFS subgroup (*rho* = –0.304, *p* = 0.034). In contrast, TSR displayed a weak positive correlation with TB, evident in the overall cohort (*rho* = 0.279, *p* = 0.010) and in the NAC subgroup (*rho* = 0.341, *p* = 0.045) (Supplementary Figure S5).

### 3.8. NAC Response in Relation to TSR, β-CTN/m-CK Index, and TB

**Figure 3:**
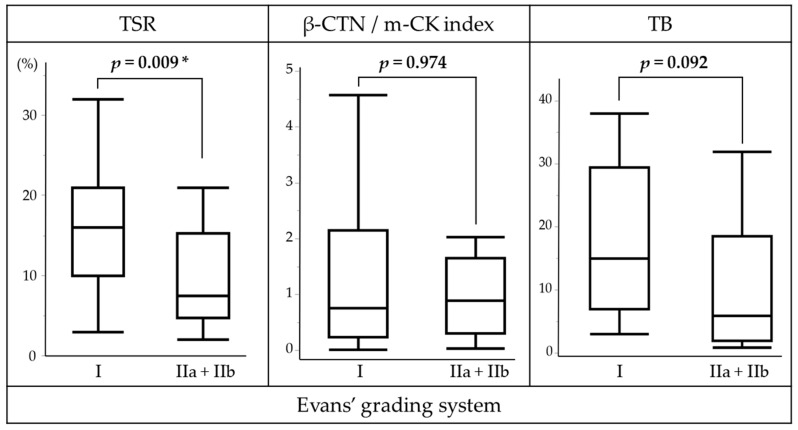
Association of TSR, β-CTN/m-CK index, and TB with histological response after NAC in PDAC. Box plots compare Evans grade I (*n* = 17; <10% tumor cell destruction) and Evans IIa (10–50%)/IIb (51–90%) grades (*n* = 18), depicting the five-number summary: minimum, first (lower) quartile, median, third (upper) quartile, and maximum values. * *p* < 0.05 was considered statistically significant. Abbreviations: TSR, tumor–stroma ratio; β-CTN, β-catenin; m-CK, multi-cytokeratin; TB, tumor budding; NAC, neoadjuvant chemotherapy; PDAC, pancreatic ductal adenocarcinoma.

In the NAC cohort, low TSR was significantly associated with a more favorable histological response. Tumors attaining Evans IIa/IIb grades (*n* = 18) exhibited a lower median TSR of 7% (interquartile range [IQR], 5–15), compared with 16% (IQR, 3–19) in Evans grade I (n = 17; *p* = 0.009). TB showed a marginal trend toward reduction in Evans IIa/IIb tumors (median 6; IQR, 2.5–16.5) relative to Evans I (median 15; IQR, 3–29; *p* = 0.092). By contrast, the β-CTN/m-CK index did not vary by Evans grade (Evans I: median 0.8, IQR 0.01–2.1; Evans IIa/IIb: median 0.9, IQR 0.3–1.6; *p* = 0.974) (Figure 3).

### 3.9. Survival Analysis in the Overall Cohort

The set of candidate prognostic variables encompassed patient factors (age, sex), treatment variables (NAC and adjuvant chemotherapy), tumor-related parameters (location, pT and pN categories, tumor differentiation, and lymphovascular, perineural, and peripancreatic fat invasion, margin status), and histopathological biomarkers (TSR, β-CTN/m-CK index, and TB). On univariate analysis, TSR ≥13% (*p* = 0.015), β-CTN/m-CK index < 0.5 (*p* = 0.009), and high TB (*p* = 0.022), together with conventional factors such as age ≥ 75 (*p* = 0.033), nodal involvement (pN1/2) (*p* = 0.022), moderate/poor differentiation (*p* < 0.001), peripancreatic fat invasion (*p* = 0.015), and absence of adjuvant chemotherapy (*p* < 0.001), were associated with worse CSS. For RFS, β-CTN/m-CK index < 0.5 (*p* < 0.001) and TSR ≥ 13% (*p* = 0.020), along with conventional variables including absence of adjuvant chemotherapy (*p* = 0.001), pT3 stage (*p* = 0.046), nodal involvement (*p* = 0.001), moderate/poor differentiation (*p* < 0.001), lymphovascular invasion (*p* = 0.004), and peripancreatic fat invasion (*p* = 0.017), were significantly associated with worse outcomes (supplementary Table S5). Multivariate models identified TSR ≥13% (HR 2.414, 95% CI 1.072–5.439; *p* = 0.034) and β-CTN/m-CK index <0.5 (HR 2.028, 95% CI 1.120–3.670, *p* = 0.020) as independent predictors of shorter CSS and RFS, respectively, together with all other conventional clinicopathological factors assessed (Table 2).

### 3.10. Survival Analysis in the NAC Subgroup

Univariate analysis identified high TB (*p* = 0.022), male sex (*p* = 0.022), moderate/poor differentiation (*p* = 0.014), and positive resection margin (*p* < 0.001) as factors associated with worse CSS, while β-CTN/m-CK index < 0.5 (*p* = 0.018) and positive margin (*p* = 0.017) were significantly related to worse RFS (Supplementary Table S6). Multivariate analysis showed positive margin (HR 63.446, 95% CI 2.871–1402.272; *p* = 0.009) and male sex (HR 18.054, 95% CI 1.614–201.926; *p* = 0.019) as independent adverse factors for CSS, with high TB also demonstrating a marginal association (HR 6.008, 95% CI 0.847–42.627; *p* = 0.073). For RFS, β-CTN/m-CK index < 0.5 (HR 2.516, 95% CI 1.031–6.138; *p* = 0.043) was the only independent adverse factor (Table 3).

### 3.11. Survival Analysis in the UFS Subgroup

On univariate analysis, both TSR ≥ 13% (CSS: *p* = 0.025; RFS: *p* = 0.020) and β-CTN/m-CK index < 0.5 (CSS: *p* = 0.032; RFS: *p* = 0.006) were significantly associated with adverse outcomes. In addition, conventional prognostic factors including moderate/poor differentiation (CSS and RFS: both *p* < 0.001), absence of adjuvant chemotherapy (CSS: *p* < 0.001; RFS: *p* = 0.009), nodal involvement (RFS: *p* = 0.004), and lymphovascular invasion (RFS: *p* = 0.036) were also significant (Supplementary Table S7). In multivariate models, β-CTN/m-CK index < 0.5 (HR 2.230, 95% CI 1.106–4.500; *p* = 0.025) was the independent predictor of shorter RFS, and TSR ≥ 13% showed marginal significance for CSS (HR 2.536, 95% CI 0.947–6.790; *p* = 0.064). In addition, conventional prognostic factors including moderate/poor differentiation (CSS: HR 7.632, 95% CI 2.471–23.575; *p* < 0.001; RFS: HR 5.006, 95% CI 2.178–11.508; *p* < 0.001), peripancreatic fat invasion (CSS: HR 4.933, 95% CI 1.104–22.033; *p* = 0.037), and absence of adjuvant chemotherapy (CSS: HR 5.539, 95% CI 1.882–16.306; *p* = 0.002) were also identified (Table 4).

**Figure 4:**
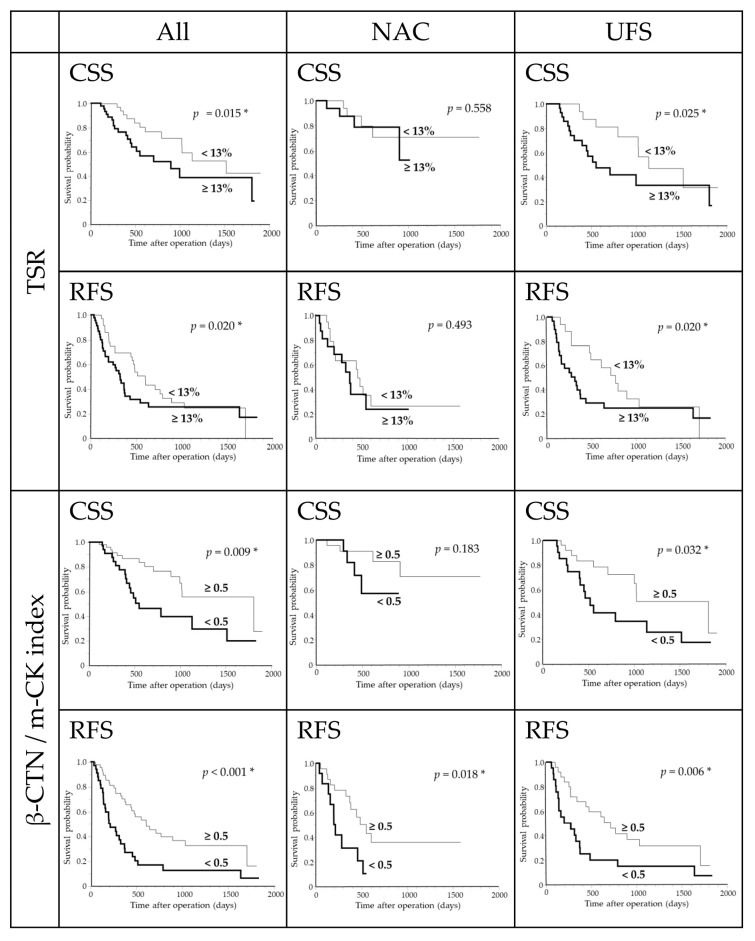
Kaplan–Meier analysis of TSR and β-CTN/m-CK index in PDAC. * *p* < 0.05 was considered statistically significant. Abbreviations: TSR, tumor–stroma ratio; β-CTN, β-catenin; m-CK, multi-cytokeratin; PDAC, pancreatic ductal adenocarcinoma; All, entire PDAC cohort; NAC, neoadjuvant chemotherapy-treated PDAC; UFS, upfront surgery-treated PDAC.

Kaplan–Meier curves for the overall PDAC cohort, NAC, and UFS subgroups stratified by TSR and β-CTN/m-CK index are shown in Figure 4. In the overall cohort, both TSR ≥ 13% and β-CTN/m-CK < 0.5 were associated with shorter CSS and RFS (both *p* < 0.05, log-rank). In the NAC subgroup, only β-CTN/m-CK < 0.5 was associated with shorter RFS (*p* = 0.018), whereas TSR showed no significant separation for either endpoint. In the UFS subgroup, TSR ≥ 13% predicted shorter CSS (*p* = 0.025) and RFS (*p* = 0.020), and β-CTN/m-CK < 0.5 likewise predicted shorter CSS (*p* = 0.032) and RFS (*p* = 0.006).

## 4. Discussion

Resection remains the standard treatment for PC, with studies suggesting potential survival benefits of NAC even in resectable disease [4,6,8,9], although others reported no clear advantage over UFS [4,5]. Although the S-1–based regimen used in Japan is less common in Western countries due to ethnic differences in tolerability, the clinical concept and benefits of NAC remain broadly applicable across chemotherapy protocols. In our cohort, survival did not differ significantly between NAC and UFS, but NAC patients tended to have lower pT stage and more frequent margin-negative resections. Further studies likewise noted lower pT stages in post-NAC specimens, particularly with stronger histological response [38,39]. These trends are consistent with prior multicenter findings using S-1 plus GEM [6,8] and reinforce the notion that NAC enhances resectability, even if survival benefits are not universally evident. The lack of statistical significance likely reflects the limited sample size and follow-up in our series.

To ensure reproducible TSR estimation, we applied a two-stage pipeline to m-CK–immunostained WSIs, combining epithelial segmentation and H&E-based tumor delineation. This approach reduces observer bias and enables objective quantification compared with conventional assessment [15,17,19,23]. High TSR (≥13%) tended to associate with margin-positive resections, consistent with prior findings that epithelial predominance correlates with invasive growth and margin involvement [14,16,19,20]. These data suggest that high TSR reflects a more aggressive invasive phenotype rather than sampling bias.

Across the entire cohort, TSR ≥ 13% independently predicted shorter CSS, with a similar trend in the UFS subgroup. This aligns with previous reports linking epithelial predominance to poor survival in PDAC [14,16,17,19]. Importantly, our study adds that digitally derived TSR preserves prognostic value even under standardized image analysis, supporting its potential as a reproducible biomarker. While abundant stroma has been associated with better outcomes [22,25], the present findings highlight that epithelial-rich tumors retain an adverse prognosis even when quantified objectively. From a clinical perspective, integrating TSR and β-catenin assessment into routine pathological workflows could enhance prognostic stratification. Digital image analysis may facilitate such implementation by providing standardized and reproducible quantification across institutions.

Within our NAC subgroup, TSR had no significant prognostic impact. Prior reports have shown that NAC modifies stromal composition, often decreasing stromal ratio or inducing encapsulating fibrosis [18,20]. Our data showed that lower TSR correlated with favorable histological response (Evans IIa/IIb), suggesting that TSR may reflect treatment efficacy rather than intrinsic biology after chemotherapy. This supports the concept that therapy-induced stromal remodeling alters the prognostic meaning of TSR. Indeed, experimental studies indicate that chemotherapy suppresses myofibroblasts and modifies stromal–epithelial interactions [26,27,52,53]. The dual nature of the stroma, which acts both as a physical barrier to drug delivery [27,53] and as a potential tumor-suppressive matrix [54], emphasizes that its biological role depends on the treatment context.

Aberrant β-CTN redistribution has been described in subsets of PDAC, but reported patterns vary across studies [31,32,33,34,35,36,37]. Discrepant nuclear staining reported previously may reflect artifacts related to antibody specificity, epitope recognition, or pre-analytical procedures [55]. Such signals should be interpreted cautiously, as they may not represent true nuclear translocation but rather nonspecific immunohistochemical artifacts. In our cohort, membranous β-CTN expression was largely preserved, with reduction mainly in poorly differentiated tumors. Stratified analysis confirmed significantly lower β-CTN/m-CK index values in these cases, supporting that de-differentiation weakens cell–cell adhesion and promotes invasion [31,32]. Unlike tumors with Wnt pathway activation, such as solid-pseudopapillary neoplasms, we found no nuclear accumulation of β-CTN, consistent with the rarity of CTNNB1 mutations in PDAC [31,56]. These findings indicate that β-CTN alterations in PDAC are primarily membranous rather than nuclear, reflecting differentiation rather than canonical Wnt activation.

To minimize variability, β-CTN was normalized to m-CK on serial sections, producing a β-CTN/m-CK index that provides internal control for staining intensity [57]. Using this metric, we compared PDACs treated with NAC or UFS and found that a low β-CTN/m-CK index (<0.5) predicted shorter RFS across all subgroups. Prior reports have shown inconsistent associations between β-CTN localization and survival [33,35,36,37], but our analysis demonstrates that decreased membranous β-CTN relative to epithelial content consistently indicates poorer outcomes, particularly after NAC. This suggests that β-CTN evaluation provides complementary information beyond histological response grading and may reflect therapy-resistant cellular phenotypes.

TB represents a well-established morphological marker of epithelial–mesenchymal transition and aggressiveness [40,41,42,43]. High TB was significantly correlated with peripancreatic fat invasion and marginally with perineural invasion, consistent with prior data [43,46,47]. In our NAC subgroup, TB was lower in Evans IIa/IIb than in Evans I tumors and correlated modestly with TSR, indicating that NAC may suppress budding activity through stromal remodeling. These observations suggest that therapeutic response reduces epithelial plasticity at the invasive front [12,20,58], supporting the potential of TSR, β-CTN/m-CK index. As TB reflects epithelial–mesenchymal transition [42,43], it may serve as a complementary morphological indicator of post-NAC tumor biology.

Conventional clinicopathological parameters also retained prognostic significance. In the NAC subgroup, margin positivity and male sex were independent adverse factors for CSS, consistent with prior reports [5,6,24]. In our UFS subgroup, moderate/poor differentiation, peripancreatic fat invasion and the absence of adjuvant chemotherapy predicted worse outcomes, consistent with previous and registry data [4,8,19]. The benefit of adjuvant therapy appeared limited to UFS patients, as NAC already selects responders, echoing findings from conversion surgery studies [49]. Future directions should include prospective validation of these findings in larger, multi-institutional and ethnically diverse cohorts, as well as evaluation of regimen-specific predictive value, particularly comparing S-1 and FOLFIRINOX (a combination of 5-fluorouracil, leucovorin, irinotecan, and oxaliplatin)-based NAC protocols. Such studies could clarify whether the observed associations between TSR, β-CTN/m-CK index, and TB remain consistent across different treatment contexts.

This study has limitations. It was a single-institution retrospective analysis with a small cohort, which may restrict generalizability. All analyses were based on resected specimens, and applicability to preoperative biopsies remains uncertain. Nevertheless, the use of standardized digital quantification enhances reproducibility and mitigates observer bias. Longer-term, multicenter validation will be needed to confirm these findings and to define how histomorphological indices such as TSR, β-CTN/m-CK, and TB can be integrated into prognostic modeling after NAC for PDAC.

## 5. Conclusions

The β-CTN/m-CK index consistently served as an independent prognostic factor across NAC-treated and UFS PDAC. TSR was associated with histological response to NAC, and TB was also evidently reduced following NAC. TSR additionally predicted cancer-specific survival in the overall cohort. Few studies have examined biomarkers capturing both prognostic relevance and therapeutic response in NAC-treated PDAC, underscoring the novelty of our findings and suggesting that integrated biomarker assessment may improve risk stratification and guide personalized treatment strategies. In UFS, adjuvant chemotherapy improved survival, and these pathological indices may help identify patients most likely to benefit from it, with deep learning-based WSI analysis offering further refinement.

## Figures and Tables

**Table 1:** Clinicopathological characteristics in patients with PDAC undergoing NAC versus UFS.

Variable	NAC	UFS	*p*-Value
Number of cases	35	49	
Age (years)			
Mean/Median (range)	69.5/71 (39–85)	74.8/75 (50–91)	0.040 *
<75	22	24	0.268
≥75	13	25	
Sex			0.664
Male	19	24	
Female	16	25	
Tumor location			1.000
Head	21	29	
Body/Tail	14	20	
Adjuvant therapy			0.264
Yes	23	25	
No	12	24	
pT category			0.060
1	13	10	
2	13	31	
3	9	8	
pN category			0.564
N0	18	21	
N1	11	15	
N2	6	13	
Tumor differentiation			0.787
Well	14	23	
Moderate	18	23	
Poor	3	3	
Lymphovascular invasion			0.468
Absent	8	15	
Present	27	34	
Perineural invasion			0.199
Absent	11	9	
Present	24	40	
Peripancreatic fat invasion			0.409
Absent	9	8	
Present	26	41	
Resection margin			0.067
Negative	33	39	
Positive	2	10	

* *p* < 0.05 was considered statistically significant. Abbreviations: PDAC, pancreatic ductal adenocarcinoma; NAC, neoadjuvant chemotherapy; UFS, upfront surgery.

**Table 2:** Multivariate analysis of clinicopathological features associated with survival outcomes in PDAC.

Variable	CSS			RFS		
	HR	95% CI	*p*-Value	HR	95% CI	*p*-Value
Adjuvant therapy (Yes vs. No)	5.395	2.233–13.031	<0.001 *	2.210	1.230–3.972	0.008 *
pT category (1 + 2 vs. 3)	N/A	N/A	N/A	1.785	0.909–3.505	0.093
pN category (N0 vs. N1 + N2)	1.477	0.651–3.350	0.350	1.953	1.093–3.492	0.024 *
Tumor differentiation (Well vs. Moderate + Poor)	7.776	2.832–21.349	<0.001 *	2.394	1.260–4.551	0.008 *
Lymphovascular invasion (Absent vs. Present)	N/A	N/A	N/A	2.546	1.218–5.321	0.013 *
Peripancreatic fat invasion (Absent vs. Present)	6.972	1.911–25.428	0.003 *	N/A	N/A	N/A
TSR (<13% vs. ≥13%)	2.414	1.071–5.439	0.034 *	N/A	N/A	N/A
β-CTN/m-CK index (≥0.5 vs. <0.5)	N/A	N/A	N/A	2.028	1.120–3.670	0.020 *

* *p* < 0.05 was considered statistically significant. Abbreviations: PDAC, pancreatic ductal adenocarcinoma; CSS, cancer-specific survival; RFS, recurrence-free survival; HR, hazard ratio; CI, confidence interval; TSR, tumor–stroma ratio; β-CTN, β-catenin; m-CK, multi-cytokeratin; N/A, not applicable.

**Table 3:** Multivariate analysis of clinicopathological features associated with survival outcomes in NAC-treated PDAC.

Variable	CSS			RFS		
	HR	95% CI	*p*-Value	HR	95% CI	*p*-Value
Sex (Female vs. Male)	18.054	1.614–201.926	0.019 *	N/A	N/A	N/A
Resection margin (Negative vs. Positive)	63.446	2.871–1402.272	0.009 *	4.033	0.817–19.922	0.087
β-CTN/m-CK index (≥0.5 vs. <0.5)	N/A	N/A	N/A	2.516	1.031–6.138	0.043 *
TB (Low vs. High)	6.008	0.847–42.627	0.073	N/A	N/A	N/A

* *p* < 0.05 was considered statistically significant. Abbreviations: NAC, neoadjuvant chemotherapy; PDAC, pancreatic ductal adenocarcinoma; CSS, cancer-specific survival; RFS, relapse-free survival; HR, hazard ratio; CI, confidence interval; β-CTN, β-catenin; m-CK, multi-cytokeratin; TB, tumor budding; N/A, not applicable.

**Table 4:** Multivariate analysis of clinicopathological features associated with survival outcomes in UFS-treated PDAC.

Variable	CSS			RFS		
	HR	95% CI	*p*-Value	HR	95% CI	*p*-Value
Adjuvant therapy (Yes vs. No)	5.539	1.882–16.306	0.002 *	2.013	0.980–4.136	0.057
Tumor differentiation (Well vs. Moderate + Poor)	7.632	2.471–23.575	<0.001 *	5.006	2.178–11.508	<0.001 *
Peripancreatic fat invasion (Absent vs. Present)	4.933	1.104–22.033	0.037 *	N/A	N/A	N/A
TSR (<13% vs. ≥13%)	2.536	0.947–6.790	0.064	N/A	N/A	N/A
β-CTN/m-CK index (≥0.5 vs. <0.5)	N/A	N/A	N/A	2.230	1.106–4.500	0.025 *

* *p* < 0.05 was considered statistically significant. Abbreviations: UFS, upfront surgery; PDAC, pancreatic ductal adenocarcinoma; CSS, cancer-specific survival; RFS, relapse-free survival; HR, hazard ratio; CI, confidence interval; TSR, tumor–stroma ratio; β-CTN, β-catenin; m-CK, multi-cytokeratin; N/A, not applicable.

## Data Availability

The data that support the findings of this study are not publicly available because they contain information that could compromise the privacy of research participants but are available from the corresponding author S.O. upon reasonable request.

## Supplementary Materials

The following supporting information can be downloaded at: https://www.mdpi.com/article/10.3390/curroncol32100578/s1, Figure S1: Correlation among β-CTN/m-CK index, TSR, and TB in PDAC, Figure S2: CONSORT-style flow diagram of case selection, Figure S3: Illustration of β-CTN/m-CK analysis for a non-neoplastic pancreatic intralobular duct in the same case shown in Figure 4, Figure S4: β-CTN/m-CK analysis in a representative high-index (≥0.5) case, Figure S5. Representative case of PDAC with TB, Table S1: Baseline patient characteristics, Table S2: Clinicopathological characteristics and their association with TSR between NAC and UFS groups in patients with PDAC, Table S3: Clinicopathological characteristics and their association with β-CTN/m-CK index between NAC and UFS groups in patients with PDAC, Table S4: Clinicopathological characteristics and their association with TB between NAC and UFS groups in patients with PDAC, Table S5: Univariate analysis of clinicopathological features associated with survival outcomes in PDAC, Table S6: Univariate analysis of clinicopathological features associated with survival outcomes in NAC-treated PDAC, Table S7: Univariate analysis of clinicopathological features associated with survival outcomes in UFS-treated PDAC.

## Abbreviations

The following abbreviations are used in this manuscript (in alphabetical order):

β-CTN	β-catenin
CI	confidence interval
CSS	cancer-specific survival
IHC	immunohistochemistry
m-CK	multi-cytokeratin
NAC	neoadjuvant chemotherapy
PC	pancreatic cancer
PDAC	pancreatic ductal adenocarcinoma
RFS	relapse-free survival
TB	tumor budding
TSR	tumor–stroma ratio
UFS	upfront surgery
WSI	whole-slide image
