# Supplementary material for: Comparative Prognostic Roles of β-Catenin Expression and Tumor–Stroma Ratio in Pancreatic Cancer: Neoadjuvant Chemotherapy vs. Upfront Surgery
Source: Curr Oncol. 2025 Oct 17;32(10):578. doi: 10.3390/curroncol32100578 (PMC12563957; doi:10.3390/curroncol32100578)
Supplement: Supplementary file 1 [file curroncol-32-00578-s001.zip › Supplementary Figures 1-4 (Cur Oncol).pdf]

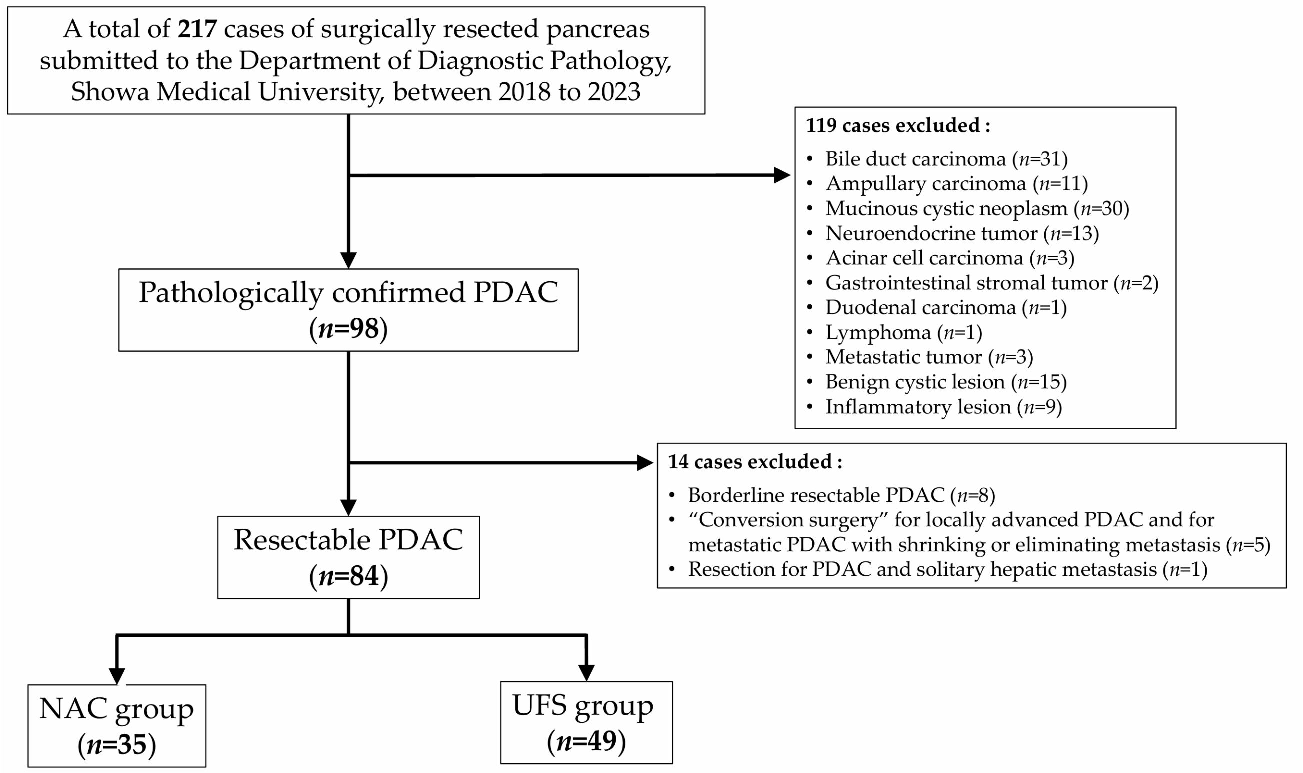

Supplementary Figure 1. CONSORT-style flow diagram of case selection. "Conversion surgery" was defined as surgical resection for locally advanced pancreatic cancer or for metastatic disease in which metastases partially regressed or completely disappeared following effective systemic chemotherapy or chemoradiation therapy. Abbreviations: PDAC, pancreatic ductal adenocarcinoma; NAC, neoadjuvant chemotherapy; UFS, upfront surgery.

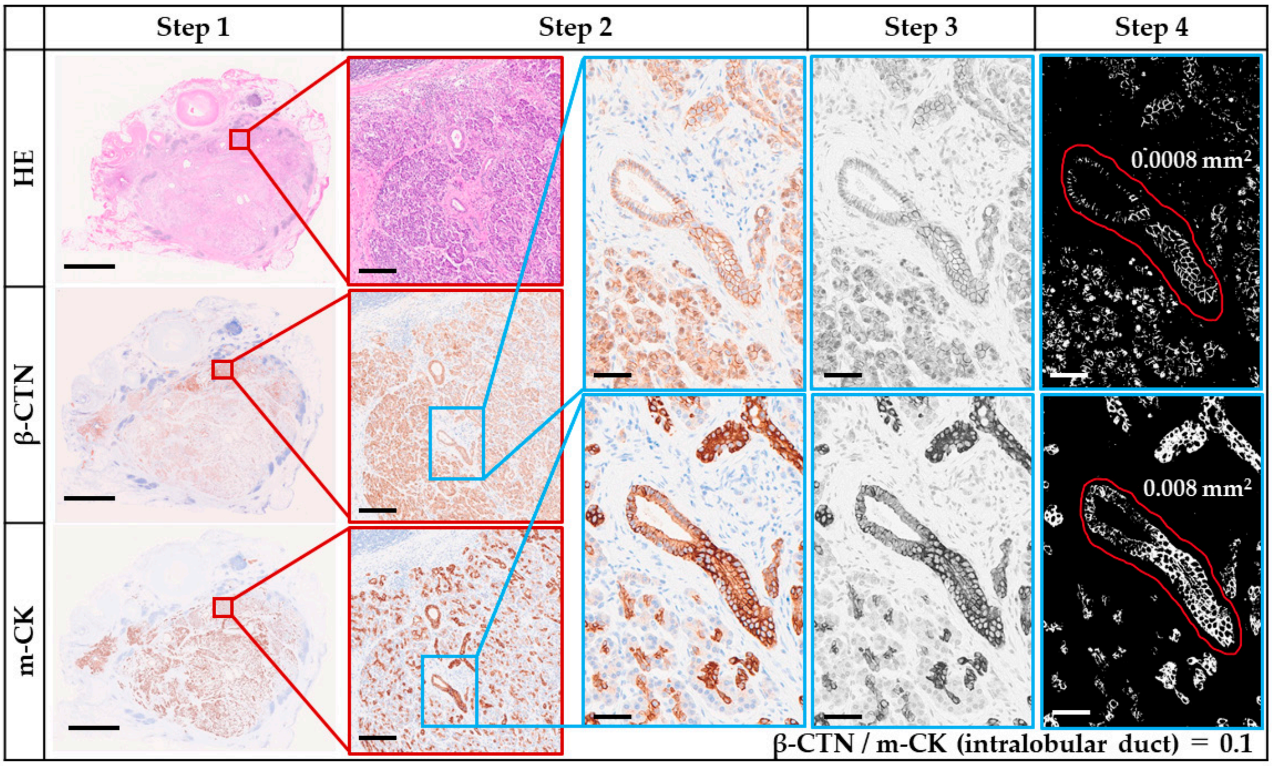

Supplementary Figure 2. Illustration of β-CTN/m-CK analysis for a non-neoplastic pancreatic intralobular duct in the same case shown in Figure 4. Step 1: Inspection of the intralobular duct using whole-slide digital images (scale bar = 5 mm). Step 2: Capture of representative intralobular duct images (scale bar = 200 μm) with higher magnification of the boxed area (scale bar = 50 μm). Step 3: Construction of gray-scale image (scale bar = 50 μm). Step 4: Construction of binary image (scale bar = 50 μm). The intralobular duct area measured by β-CTN was 0.0008 mm^2, by m-CK was 0.008 mm^2, yielding a β-CTN/m-CK ratio of 0.1 for the non-neoplastic intralobular duct. Abbreviations: β-CTN, β-catenin; m-CK, multi-cytokeratin.

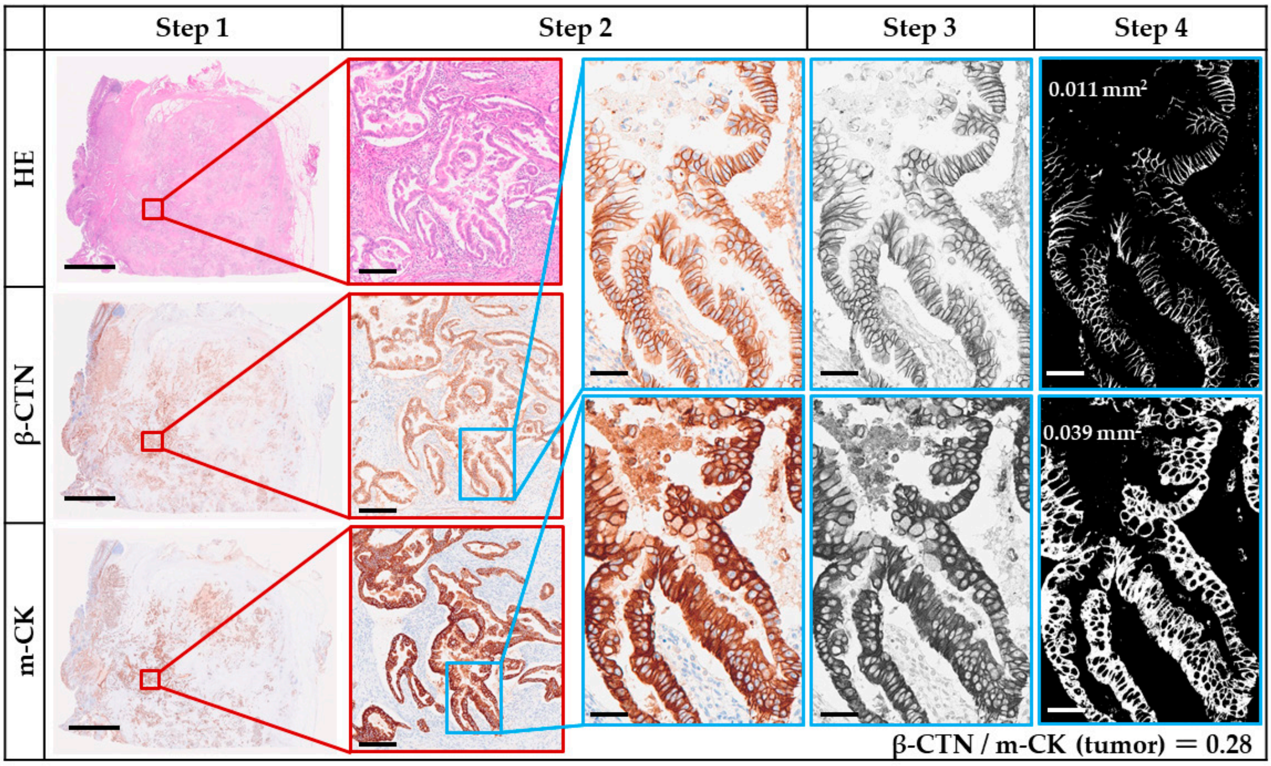

Supplementary Figure 3. β-CTN/m-CK analysis in a representative high-index (≥ 0.5) case, performed using the same stepwise procedure (Steps 1–4) as in Figure 2. Tumor area measured 0.011 mm^2 in β-CTN images and 0.039 mm^2 in m-CK images, corresponding to a β-CTN/m-CK ratio of 0.28. In the pancreatic intralobular duct, the ratio was 0.08. Based on these values, the final β-CTN/m-CK index was calculated as 3.5. Abbreviations: β-CTN, β-catenin; m-CK, multi-cytokeratin.

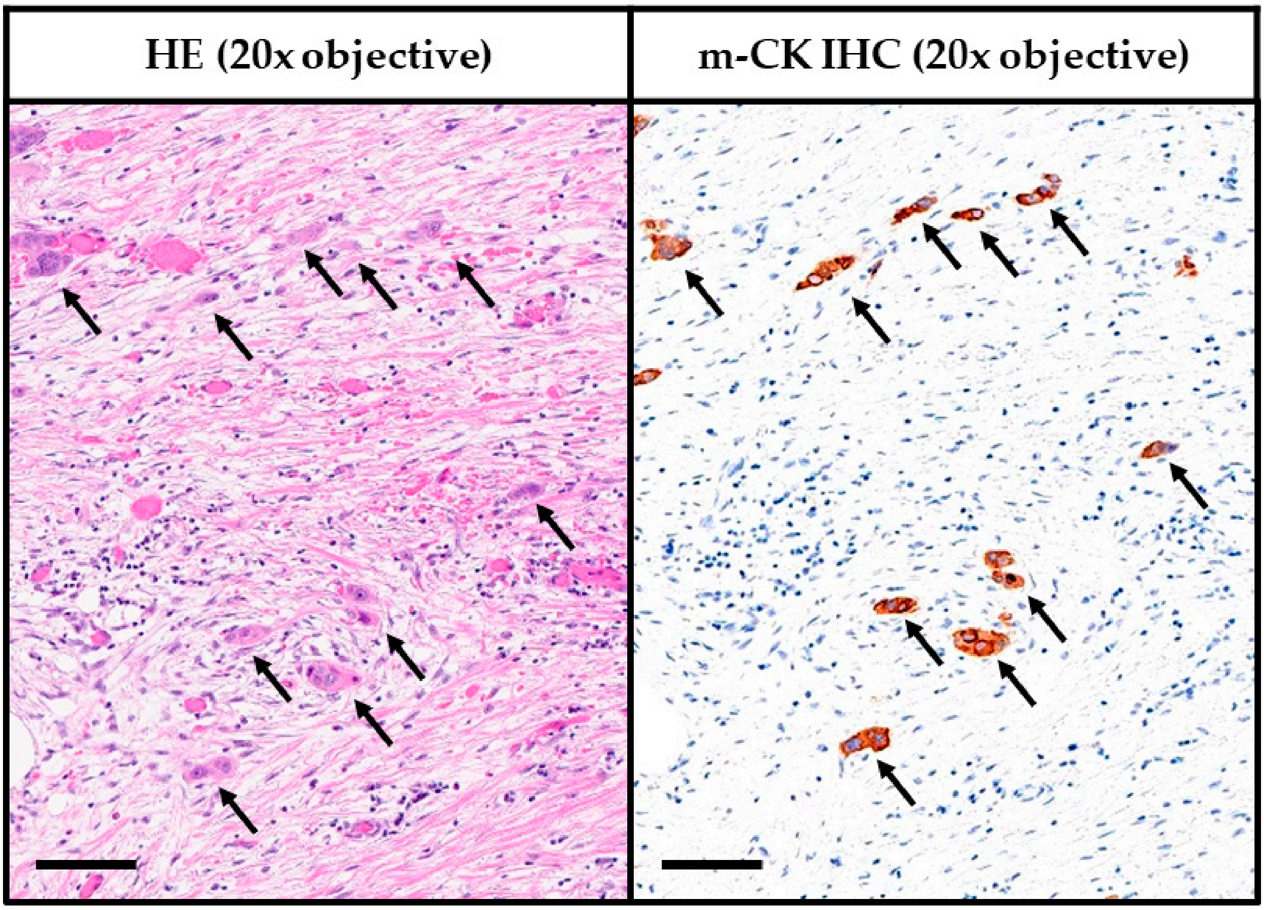

Supplementary Figure 4. Representative case of PDAC with TB (*arrows*; scale bar = 100 μm). Abbreviations: PDAC, pancreatic ductal adenocarcinoma; TB, tumor budding; HE, hematoxylin and eosin staining; m-CK IHC, immunohistochemistry for multi-cytokeratin.

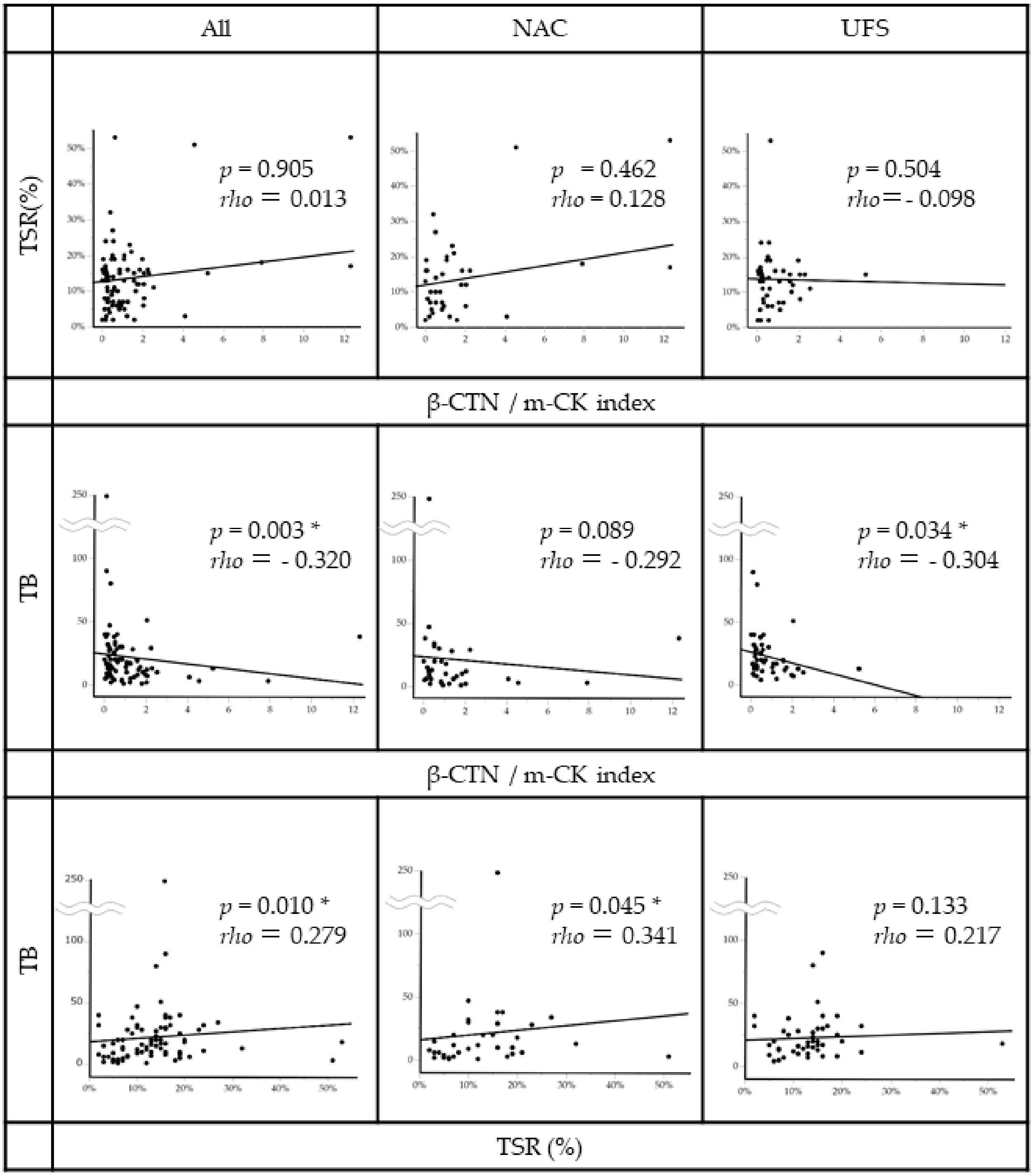

Supplementary Figure 5. Correlation among β-CTN/m-CK index, TSR, and TB in PDAC. * *p*<0.05 was considered statistically significant. Abbreviations: β-CTN, β-catenin; m-CK, multi-cytokeratin; TSR, tumor-stroma ratio; TB, tumor budding; PDAC, pancreatic ductal adenocarcinoma; All, entire PDAC cohort; NAC, neoadjuvant chemotherapy–treated PDAC; UFS, upfront surgery–treated PDAC.
