# Supplementary material for: Comparative Prognostic Roles of β-Catenin Expression and Tumor–Stroma Ratio in Pancreatic Cancer: Neoadjuvant Chemotherapy vs. Upfront Surgery
Source: Curr Oncol. 2025 Oct 17;32(10):578. doi: 10.3390/curroncol32100578 (PMC12563957; doi:10.3390/curroncol32100578)
Supplement: Supplementary file 1 [file curroncol-32-00578-s001.zip › supplementary Methods (Oikawa, et al).pdf]

Supplementary Methods: Detailed workflow for TSR quantification using ImageJ

ImageJ software (National Institutes of Health, Bethesda, MD, USA), a freely available open-source platform, was used to quantify tumor–stroma ratio (TSR). Digitalized whole-slide images (WSIs) of multi-cytokeratin (m-CK) immunohistochemistry were captured at ×40 magnification and saved in JPEG format.

Calibration

Each JPEG file was opened in ImageJ (“File > Open”).

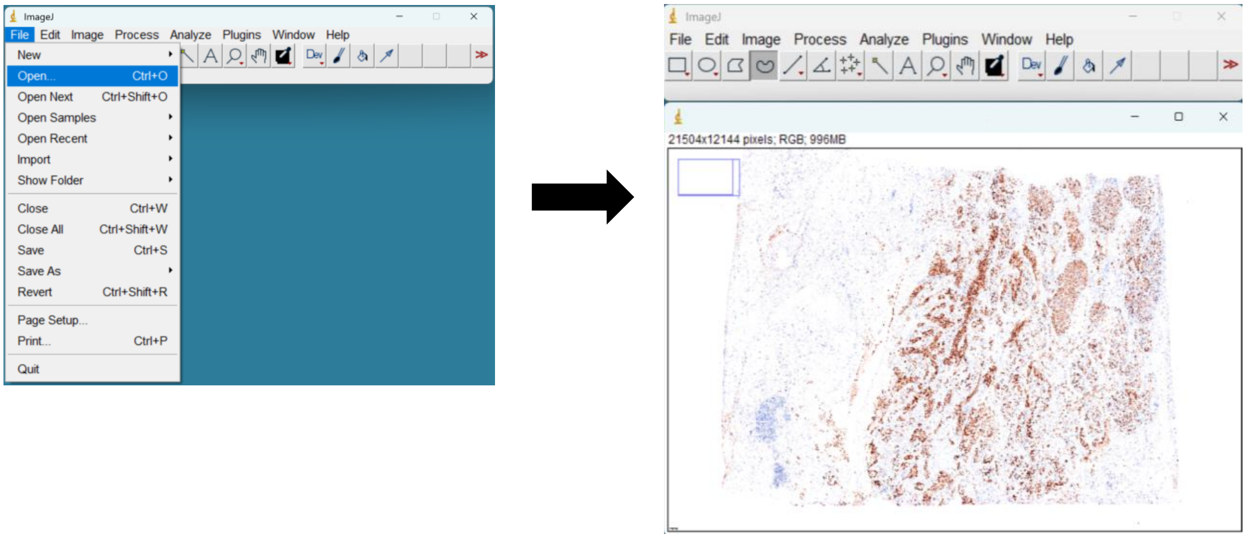

The scale was calibrated by drawing a line along the reference scale bar using the “Straight Line” tool (*yellow line, indicating by red arrow*) and selecting “Analyze > Set Scale.” The known distance (0.5) and unit of length (mm) were entered accordingly (*red boxes*).

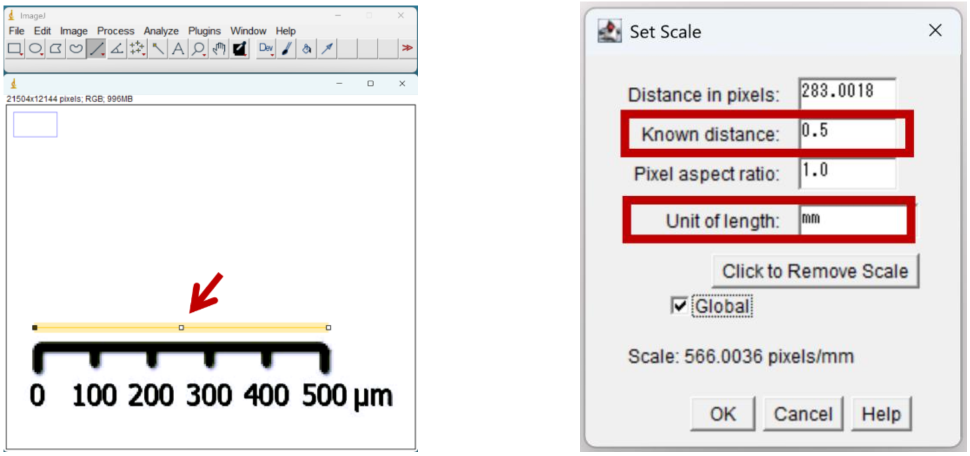

Tumor annotation

The tumor outline was manually traced using the "Freehand Selections" tool, and non-tumor areas outside the margin were removed with "Edit > Clear Outside."

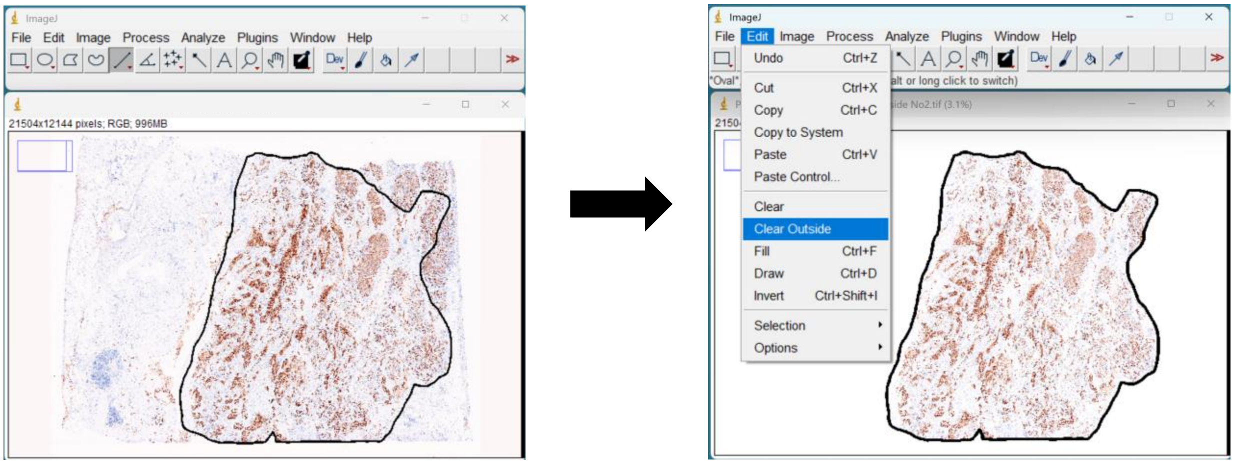

Within the tumor, non-neoplastic pancreatic parenchyma was delineated using the same tool and registered in the ROI Manager ("Analyze > Tools > ROI Manager"). All non-tumorous regions were then summed (*No. 1–39, highlighted by the red box*) within the tumor mass. These areas were subsequently subtracted from the area of the entire

tumor mass (*No. 40, highlighted by the light blue box*), which originally included cancer tissue, stroma, and non-neoplastic pancreatic parenchyma. The final calculated area (mm^2) of cancer and stroma, obtained by subtracting the summed non-neoplastic regions (*the red box*) from the entire tumor mass (*the light blue box*), represented the combined cancer and stroma area, which served as the reference for subsequent calculation of stromal area and TSR.

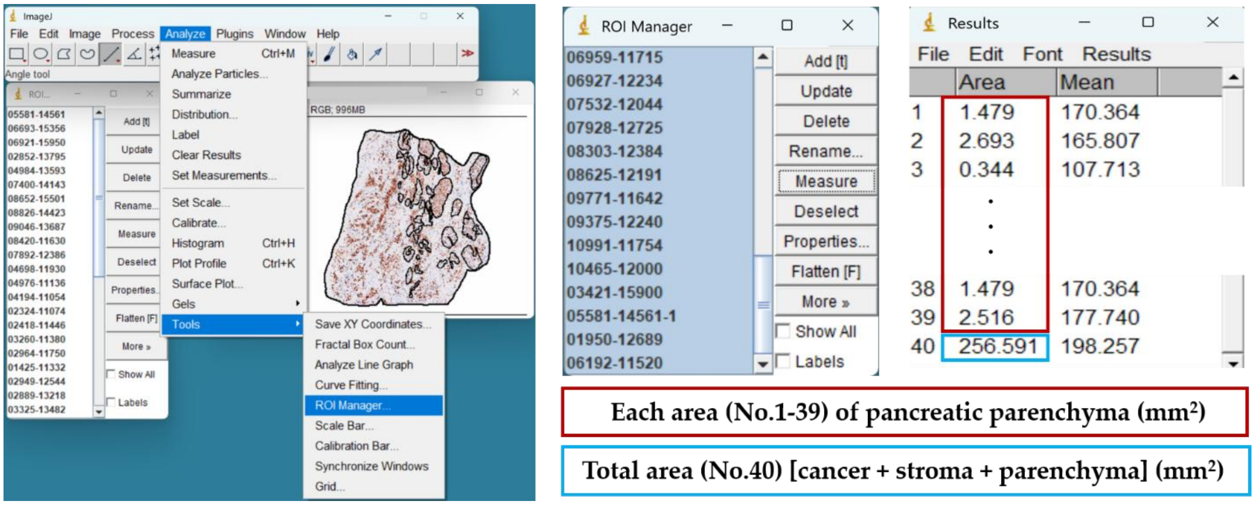

Grayscale conversion and thresholding

The corrected image was converted to 8-bit grayscale ("Image > Type > 8-bit") followed by thresholding ("Image > Adjust > Threshold").

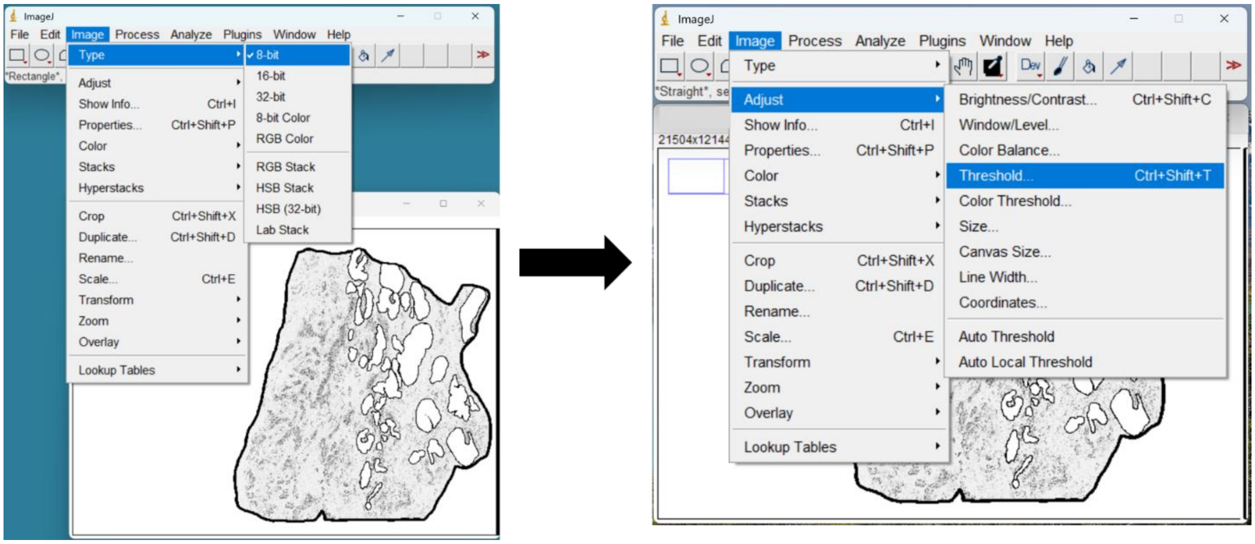

The minimum threshold was set to 0 and the maximum fixed at 140 for all cases (*red box*). Applying this threshold generated a binary image in which m-CK–positive tumor epithelium appeared as white particles, while the surrounding stroma was represented as black. Non-neoplastic parenchymal islands previously excluded were not visible in the binary image (*yellow box shows a magnified view of the binary image*).

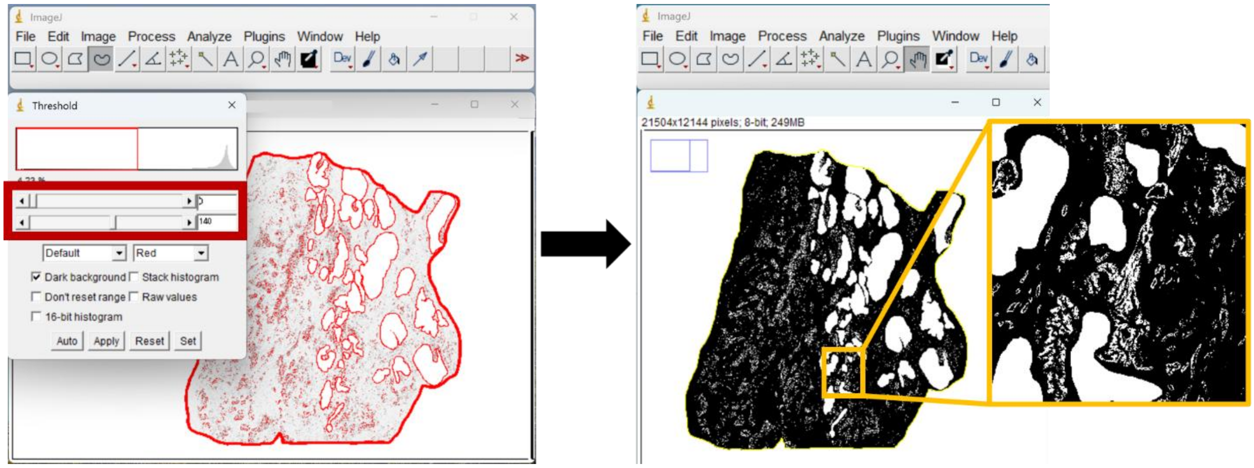

Quantification of tumor epithelium and stroma

Tumor areas (white pixels) were quantified using "Analyze > Analyze Particles" with both "Display Results" and "Summarize" options enabled. In "Summary", total area (mm^2) is consistent with cancer area (*red box*); for example, in one representative case, the cancer area measured 17.662 mm^2. The stromal area was then determined by subtracting the cancer area from the final calculated area of cancer and stroma, as obtained in the preceding *tumor annotation* step.

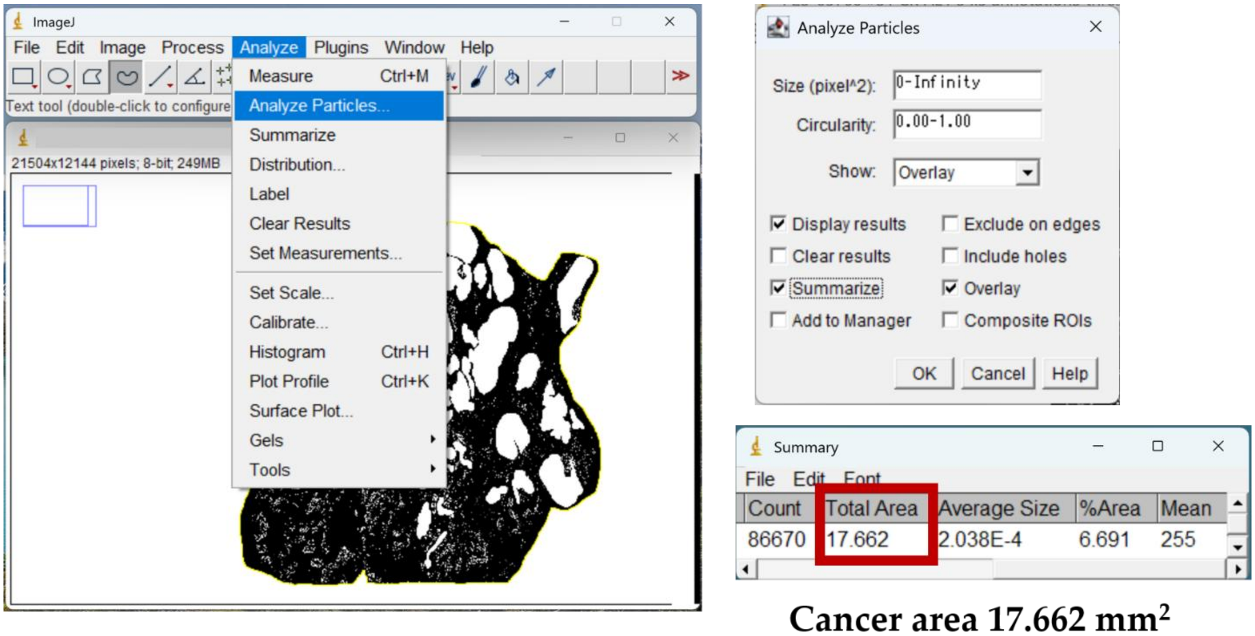

Cancer area 17.662 mm^2

Calculation of TSR

The final calculated area (cancer + stroma) corresponds to the denominator in the TSR formula (i.e., m-CK–positive tumor area + m-CK–negative stromal area). TSR was therefore calculated as:

$$TSR\ (\%) = \frac{m\text{-}CK\text{–}positive\ tumor\ area\ [mm^2]}{m\text{-}CK\text{–}positive\ tumor\ area\ + m\text{-}CK\text{–}negative\ stromal\ area\ [mm^2]}\ X100$$

Quality control

Annotations were initially performed by one investigator (S.O.) and independently reviewed by a board-certified pathologist (H.M.) to ensure reproducibility.
